# Study of the tumor microenvironment during breast cancer progression

**DOI:** 10.1186/s12935-017-0492-9

**Published:** 2017-12-22

**Authors:** Rahil Eftekhari, Rezvan Esmaeili, Reza Mirzaei, Katayoon Bidad, Stacy de Lima, Maryam Ajami, Hedayatollah Shirzad, Jamshid Hadjati, Keivan Majidzadeh-A

**Affiliations:** 1 Department of Immunology, School of Medicine, Tehran University of Medical Sciences, Tehran, Iran; 2 Genetics Department, Breast Cancer Research Center, Motamed Cancer Institute, ACECR, Tehran, Iran; 3 Immunology, Asthma and Allergy Research Institute, Tehran University of Medical Sciences, Tehran, Iran; 4 Inflammation Research Network-Snyder Institute for Chronic Disease, Department of Physiology and Pharmacology, University of Calgary Cumming School of Medicine, Calgary, AB Canada; 5 Department of Immunology, Faculty of Medical Sciences, Tarbiat Modares University, Tehran, Iran; 6 Medical Plants Research Center, Shahrekord University of Medical Sciences, Shahrekord, Iran

**Keywords:** Breast cancer, Inflammation, Microenvironment, T helper

## Abstract

**Background:**

Different cells and mediators in the tumor microenvironment play important roles in the progression of breast cancer. The aim of this study was to determine the composition of the microenvironment during tumor progression in order to discover new related biomarkers and potentials for targeted therapy.

**Methods:**

In this study, breast cancer biopsies from four different stages, and control breast biopsies were collected. Then, the mRNA expression of several markers related to different CD4^+^ T cell subsets including regulatory T cells (Treg), T helper (Th) type 1, 2 and 17 were determined. In addition, we investigated the expression of two inflammatory cytokines (TNF-α and IL-6) and inflammatory mediators including FASL, IDO, SOCS1, VEGF, and CCR7.

**Results:**

The results showed that the expression of Th1 and Th17 genes was decreased in tumor tissues compared to control tissues. In addition, we found that the gene expression related to these two cell subsets decreased during cancer progression. Moreover, the expression level of TNF-α increased with tumor progression.

**Conclusion:**

We conclude that the expression of genes related to immune response and inflammation is different between tumor tissues and control tissues. In addition, this difference was perpetuated through the different stages of cancer.

## Background

Breast cancer is a major health burden worldwide, and the primary cause of cancer-related death in women [1, 2]. It affects more than 1 million women globally, and is responsible for more than 400,000 deaths annually [2, 3]. Cancer progression is a complicated process involving immune–tumor cell interactions through numerous molecular and cellular factors within the tumor microenvironment [4]. The tumor microenvironment is comprised of a diverse milieu of cytokines, growth factors, tumor and immune cells, compounded with anti-tumor functions—mostly in response to tumor-derived signals—suppressed in tumor surroundings [5]. Several reports have linked the presence or absence of certain cell types in the tumor microenvironment with tumor stages, prognosis, and/or patient survival [6–8].

Immune cells are one of the most important players in the tumor microenvironment which include different types of leukocytes, one of most interest being the T cell [9]. It has been revealed that infiltrating T cells and production of cytokines into tumor tissue is associated with improved clinical outcome in numerous types of cancers [7, 10]. More specifically, T helper (Th) cells play central roles in the development of immune responses [11]. Recent studies have indicated that the balance between different CD4^+^ Th subsets (Th1, Th2, Th17, and Treg) is important in anti-tumor immunity, and perhaps, in the process of tumor progression [12, 13]. Furthermore, it has been shown that the density and type of immune cells as well as various inflammatory factors greatly influence cancer growth [14–16]. In recent studies, the association between tumor progression, metastasis and inflammatory mediators TNF-α, IL-6, vascular endothelial growth factor (VEGF) and C-C motif chemokine receptor 7 (CCR7) have also been investigated [17–19]. Collectively, it is still under investigation whether the immunological patterns are a better predictor of patient survival rather than the histopathological methods presently used for cancer staging. Nevertheless, accumulating data does support the hypothesis that the composition of tumor microenvironment influences the metastatic events and survival of patients.

This study aimed to investigate the presence of different CD4^+^ T cell subsets (Th1, Th2, Th17, and Treg), the inflammatory cytokines (IL-6 and TNF-α) as well as some other markers which play roles in immune cell functions including IDO (Indoleamine 2,3-dioxygenase), Fas ligand, SOCS1 (suppressor of cytokine signaling 1), VEGF and CCR7. The expression of all these factors was measured in different stages of breast cancer and were compared against normal breast tissues.

## Methods

### Patients

In the current study, biopsies of untreated breast cancer patients (n = 54) and control breast tissues from apparently healthy subjects referring for aesthetic surgeries (n = 11) were obtained from the Breast Cancer Research Center Biobank (BCRC-BB) in Iran (Table 1). According to the protocols followed by this bank, immediately after excisional biopsy or surgery, sample tissues were snap-frozen in liquid nitrogen and stored at − 70 °C. The content of cancer cells in each sample was pathologically checked and the tissues with less than 80% tumor area were eliminated from the study. ICBC-BB is obliged to ethical guidelines and recommendations for biobanks on the storage and use of human biological samples.

**Table 1:** Clinicopathological features

Parameters	Normal	Stage I	Stage II	Stage III	Stage IV
Number of cases	11	14	15	15	10
Age (years) (mean ± SEM)	40 ± 2.6	48 ± 2	53 ± 3	43 ± 2.6	55 ± 3.5
Menopausal status					
Pre	84	84.6	55.5	73	62.5
Post	16	15.4	44.5	27	37.5
Type					
IDC	NA	100	93	87.5	100
ILC	NA	0	7	12.5	0
Hormone status					
ER Positive	NA	77	53	80	62.5
PR Positive	NA	54	44	80	50
Her2/neu positive	NA	23	37.5	53	37.5
p53 positive	NA	70	30	33	33

All values except number of cases and age represent N (%)

*NA* not applicable, *IDC* invasive ductal carcinoma, *ILC* invasive lobular carcinoma, *ER* estrogen receptor, *PR* progesterone receptor, *Her2/neu* human epidermal growth factor receptor 2

Written informed consent was obtained from all patients and controls. The patient cohort was 32–75 years of age, with pathologically confirmed breast cancer with clinical staging according to the TNM method (tumor size, lymph node involvement, and distant metastasis). Stage groupings were based on the American Joint Committee on Cancer (AJCC). Estrogen receptor (ER), progesterone receptor (PR), human epidermal growth factor receptor 2 (Her2/neu) and p53 status are based on immunohistochemistry (IHC) results (Table 1).

### RNA extraction and cDNA synthesis

Frozen tissues (10–15 mg) while keeping on dry ice were homogenized. RNA was isolated using the RNeasy mini kit (QIAGEN) according to the manufacturer’s instructions. The concentration of extracted RNA was quantified by spectrophotometer (Hitachi, U-0080D, Japan) and we used ratio at 260/280 and 260/230 to control the purity of RNA. Extracted RNA was used for cDNA synthesis using the QuantiTect reverse transcription kit (QIAGEN). RNA extraction and cDNA synthesis was performed for each sample and then fold change was calculated for individual samples separately.

### Quantitative reverse-transcription PCR primers

Specific mRNA sequences of studied genes (Table 2) were acquired from the public GenBank sequence database of the National Center for Biotechnology Information (http://www.ncbi.nlm.nih.gov). Subsequently, all primers were designed using Gene Runner v.3.05 and confirmed with primer express 3.0. In conventional PCR, all primers generated only one amplification band visualized by agarose gel electrophoresis, indicating specificity. In this study, TFRC (transferrin receptor) and ACTB (β-actin) were considered as housekeeping genes, because of the most stable expression in breast tissue [20].

**Table 2:** List of primers

No.	Gene code	Forward	Melting point of amplicon
		Reverse	
1	T.bet	5′CCAACAATGTGACCCAGATGATT3′	85
		5′TATGCGTGTTGGAAGCGTTG3′	
2	IL-12p40	5′GCCCAGAGCAAGATGTGTCA3′	82
		5′GGGCATCCGGATACCAATC3′	
3	IFN-γ	5′TCAGCTCTGCATCGTTTTGG 3′	79
		5′GTTCCATTATCCGCTACATCTGAA3′	
4	GATA3	5′CCCTACTACGGAAACTCGGTCA3′	88
		5′GTAGGGATCCATGAAGCAGAGG3′	
5	IL-4	5′CAAGCAGCTGATCCGATTCC3′	81
		5′TTCTCTCTCATGATCGTCTTTAGCC3′	
6	IL-5	5′CCTGTTCCTGTACATAAAAATCACCA3′	80
		5′TTGAATAGTCTTTCCACAGTACCCC3′	
7	RORC	5′ACAGCACCGAGCCTCACG3′	85
		5′CAGACGACTTGTCCCCACAGA3′	
8	IL-17	5′TTGATTGGAAGAAACAACGATGACT3′	81
		5′TGGATTTCGTGGGATTGTGAT3′	
9	CCL22	5′TGCCGTGATTACGTCCGTTA3′	87
		5′CGGCACAGATCTCCTTATCCC3′	
10	FOXP3	5′ACAGCACATTCCCAGAGTTCCT3′	82
		5′GATGAGCGTGGCGTAGGTG3′	
11	CTLA4	5′TGGATCCTTGCAGCAGTTAGTTC3′	79
		5′CATTTTCACATAGACCCCTGTTGTA3′	
12	IL-13	5′AGGTCTCAGCTGGGCAGTTTT3′	80
		5′TAATGATGCTTTCGAAGTTTCAGTTG3′	
13	STAT3	5′CTCAAGAGTCAAGGAGACATGCA3′	85
		5′CTCACTCACGATGCTTCTCCG3′	
14	FASL	5′CTCCGAGAGTCTACCAGCCAGAT3′	84
		5′CATGGACCTTGAGTTGGACTTG3′	
15	CCR7	5′GTGGTGGCTCTCCTTGTCATTT3′	84
		5′ATGATAGGGAGGAACCAGGCTT3′	
16	IL-6	5′CCTGAGAAAGGAGACATGTAACAAGAG3′	81
		5′GCAAGTCTCCTCATTGAATCCAG3′	
17	VEGF	5′AGGAGGAGGGCAGAATCATCA3′	81
		5′CTCGATTGGATGGCAGTAGCT3′	
18	IL-10	5′GTGATGCCCCAAGCTGAGA3′	86
		5′CACGGCCTTGCTCTTGTTTT3′	
19	TGF-β	5′CCTGGACACCAACTATTGCTTCA3′	83
		5′TGCGGAAGTCAATGTACAGCTG3′	
20	IDO1	5′CTCTGCCAAATCCACAGGAAA3′	79
		5′TCTCAACTCTTTCTCGAAGCTGG3′	
21	SOCS1	5′CCCTGGTTGTTGTAGCAGCTTAA3′	80
		5′GGTTTGTGCAAAGATACTGGGTATATG3′	
22	TNF-α	5′CCCAGGGACCTCTCTCTAATCA3′	84
		5′ATGGGCTACAGGCTTGTCACTC3′	
23	ACTB	5′CAGCAGATGTGGATCAGCAAG3′	83
		5′GCATTTGCGGTGGACGAT3′	
24	TFRC	5′ACCGGCACCATCAAGCT3′	80
		5′TGATCACGCCAGACTTTGC3′	

Melting point of amplicon is calculated by melt curve analysis using SDS software v2.6 Applied Biosystems

### Quantitative reverse-transcription PCR

Quantitative reverse-transcription PCR (qPCR) was carried out in the 96-well plate for each sample using precision ™2X qPCR Mastermix (PrimerDesign Ltd, UK) in 20 μL reactions. The mRNA expression levels were detected by qPCR on the StepOnePlus™ system (Applied Biosystems) using incorporation of SYBR green fluorescent dye into the double-stranded PCR products. The expression level of each gene was normalized to the expression level of ACTB and TFRC housekeeping genes. CT values for each product were determined to calculate 2^−ΔΔCT^ referenced to normal breast tissues.

### Statistical analysis

The results are expressed as mean ± standard error of mean (SEM). For multiple group, a 1-way ANOVA with Tukey’s post hoc comparisons was used. P value < 0.05 was considered significant. All analysis was done using Graph Pad Prism 3.02 (GraphPad Software, San Diego, Calif).

## Results

### Expression of Th1 subset markers

**Fig. 1:**
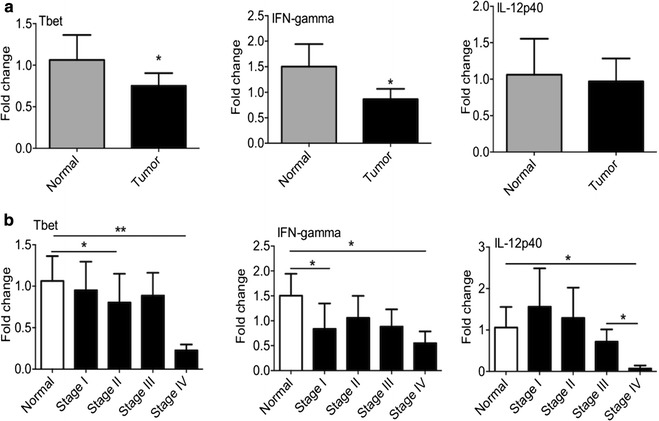
The expression of genes related to T helper 1 cells. All samples were collected from breast cancer patients and control group. RNA was extracted and the expression of genes including T-bet (Th1 cells transcription factor), IFN-γ (signature cytokine of Th1 cells) and IL-12p40 (the most important cytokine in induction of Th1 cells) was measured by qPCR. The expression level of above genes **a** in breast cancer and normal tissues **b** in different stages of tumor microenvironment and control breast biopsies was determined. *Pv < 0.05, **Pv < 0.01

To clarify the role of Th1 cells in tumor progress, the expression of Th1 subset markers was investigated in different stages of tumor microenvironment and control breast biopsies. These markers include: T-bet (Th1 cells transcription factor), IFN-γ (signature cytokine of Th1 cells), and IL-12p40 (the most important cytokine in induction of Th1 cells). As demonstrated in Fig. 1a, the presence of Th1 cells identified by T-bet expression and IFN-γ in the microenvironment of breast cancer tissues was decreased compared to the microenvironment of control breast tissue. As shown in Fig. 1b, T-bet and IFN-γ had decreasing patterns in breast cancer tissues, reaching statistical significance for T-bet in stages II and IV and for IFN-γ in stages I and IV compared to control tissues. IL-12p40 expression showed a decreasing model in the progression of tumor reaching statistical significance in stage IV samples compared to normal and stage III samples.

### Expression of Th2 subset markers

**Fig. 2:**
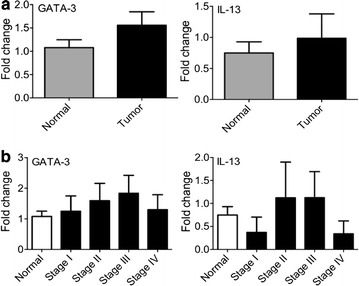
The expression of T helper 2 subset markers. RNA was extracted from breast cancer and normal tissues. The expression level of GATA-3 (principle transcription factor of Th2 cells) and IL-13 was measured by qPCR. The amount of above genes was determined **a** in the breast cancer and normal tissues **b** in the different stages of tumor and control breast biopsies

The presence of Th2 cells was investigated by measuring the level of GATA-3 (principle transcription factor of Th2 cells), IL-13, IL-4 and IL-5 (the major cytokines released by Th2 cells). Based on the results, we could not find IL-4 and IL-5 expression by qPCR up to the 40th cycle. There was no difference in the expression of GATA-3 or IL-13 genes between normal and tumor tissues and also tumor biopsies of different stages (Fig. 2a, b).

### Expression of Th17 subset markers

**Fig. 3:**
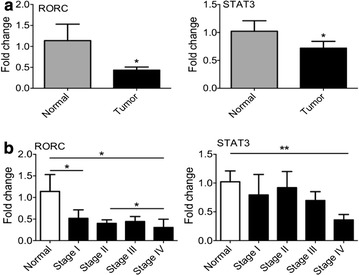
The evaluation of T helper 17 cells related genes. RNA was extracted from breast cancer and normal samples. The expression of RORC (main transcription factor of Th17 cells) and STAT3 (Signal transducer and activator of transcription 3 (STAT3) was measured by qPCR. The quantity of genes was determined **a** in the breast cancer and normal biopsies **b** in the different stages of tumor and control breast tissues. *Pv < 0.05, **Pv < 0.01

To compare the expression of Th17 genes in the microenvironment of breast cancer, RORC and STAT3 (main transcription factors of Th17 cells) and IL-17A (the signature cytokine of Th17 cells) were measured. The amount of IL-17A mRNA could not be measured by qPCR up to the 40th cycle. As demonstrated in Fig. 3a, the expression level of the RORC and STAT3 was significantly decreased in breast cancer tissues compared to control samples. The results of our study showed that the expression of RORC declined during tumor progression. The amount of RORC was significantly reduced in stage I and IV compared to control. Similarly, STAT3 expression was meaningfully decreased in the last stage of breast cancer.

### Expression of Treg subset markers

**Fig. 4:**
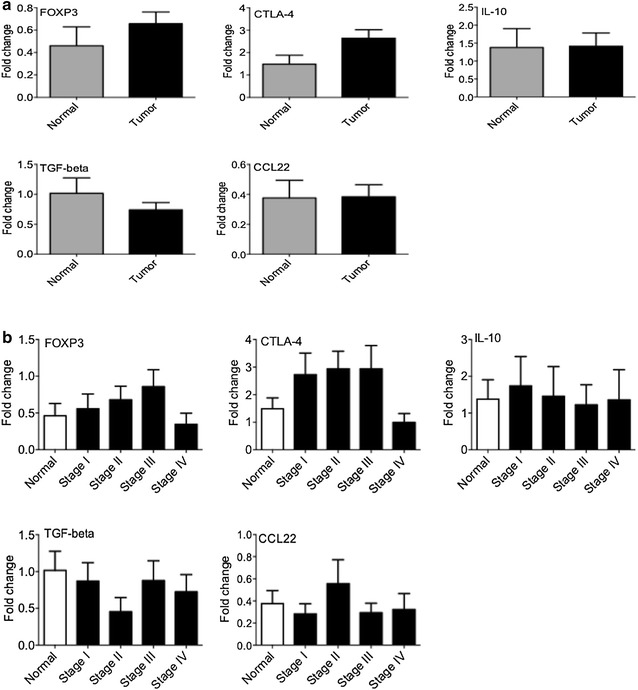
The measurement of regulatory T cells (Treg) major markers. RNA was extracted from different stages of breast cancer and control tissue. As main markers of Treg cells FOXP3 (a transcription factor which is mainly related to Treg cell subsets), TGF-β and IL-10 (Treg cell cytokines), CCL22 (a chemokine ligand which plays role in Treg cell migration) and CTLA-4 (an inhibitory receptor on Treg cells) were analyzed by qPCR. The level expression of above genes was determined **a** in the breast cancer and normal biopsies **b** in the different stages of tumor and normal breast tissues

The expression of the major markers of Treg cells, including FOXP3 (a transcription factor which is mainly related to Treg cell subsets), IL-10 and TGF-β (the signature cytokines of these cells), CCL22 (a chemokine ligand which is involved in Treg cell migration) and CTLA-4 (an inhibitory receptor which is dominantly expressed on Treg cells) in tumor tissue was determined. As shown in Fig. 4a, b, there was no significant difference in the expression of Treg cell markers between tumor and control groups and among different tumor stages.

### The relationship between hormone receptor, Her2/neu, p53 status and T cell subsets

**Fig. 5:**
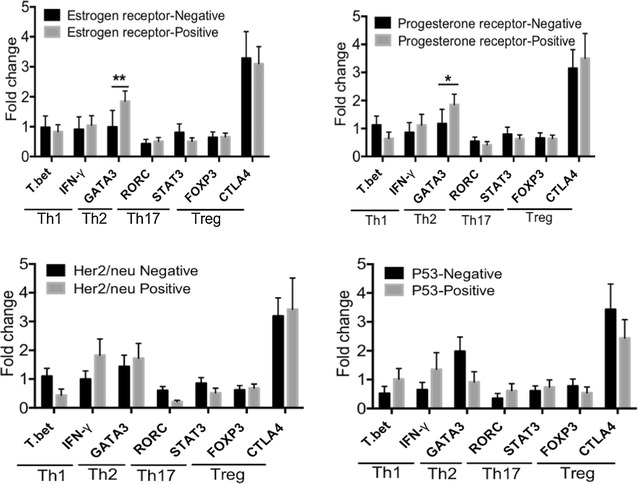
The association between T cell subsets and expression/absence of hormone receptors, Her2/neu and p53 in tumor cells. RNA was extracted from breast cancer and normal tissues; main markers of T cell subsets; Th1 (T.bet, IFN-γ), Th2 (GATA3), Th17 (RORC, STAT3) and Treg (FOXP3, CTLA4) were analyzed by qPCR. The expression of estrogen receptor (ER), progesterone receptor (PR), Her2/neu and p53 status are based on IHC results. *Pv < 0.05, **Pv < 0.01

Following analysis of Th1, 2, 17 and Treg markers, their expressions in respect to the status of PR, ER, Her2/neu and p53 in patients were investigated. The results show that there is significantly higher expression of GATA3 (Th2 marker) in ER and PR positive patients than negative patients (Fig. 5). This association was not found with any other markers.

### Expression of inflammatory cytokines

**Fig. 6:**
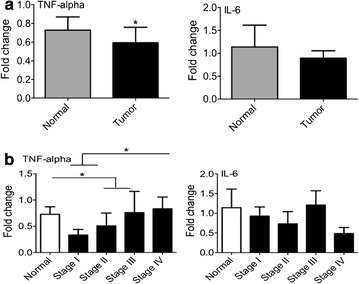
The expression of inflammatory cytokines. To survey the amount of inflammation in microenvironment of breast cancer and normal tissues, two principal inflammatory cytokines, TNF-α and IL-6 were analyzed. From different stages of breast cancer and control group, RNA was extracted, then the level of gene expression was measured by qPCR. **a** In the tumor and normal breast tissues **b** in the different stages of breast cancer and normal group. *Pv < 0.05, **Pv < 0.01

In the present study, two chief inflammatory cytokines, TNF-α and IL-6 were analyzed. We found that the expression of TNF-α was lower in the microenvironment of tumor tissue compared to control tissue (Fig. 6a). The data signified that TNF-α was decreased in stage II compared to control biopsies. In addition, there is an augmenting pattern in breast cancer stages with statistical significance in stage IV compared to stages I and II and between stage III and control group (Fig. 6b). IL-6 expression was not statistically different in tumor tissues compared to control samples (Fig. 6a, b).

### Expression of markers involved in tumor progression

**Fig. 7:**
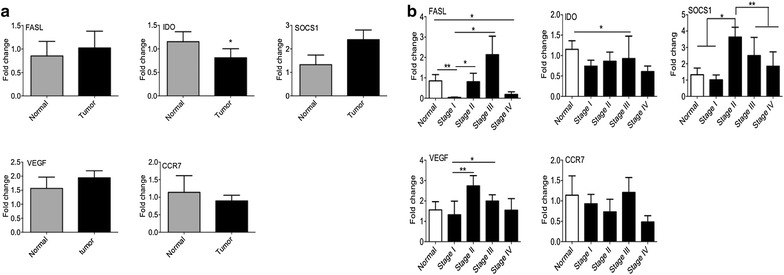
The expression of tumor progression markers. RNA was extracted from different stages of breast cancer tissues and normal group. The expression levels of inhibitory markers including FASL, IDO, SOCS1, VEGF, and CCR7 were determined by qPCR. **a** The mRNA concentration of above factors was compared between tumor and normal subjects. **b** Moreover, we investigated the expression levels of genes in different stages of patients versus control. *IDO* indoleamine 2,3-dioxygenase, *FASL* fas ligand, *VEGF* vascular endothelial growth factor, *SOCS1* suppressor of cytokine signaling 1, *CCR7* C-C motif chemokine receptor 7. *Pv < 0.05, **Pv < 0.01

In order to determined role of some factors which are involved in cancer progression IDO, FASL, VEGF, SOCS1 and CCR7 were measured. Although, there were no significant differences in expression of FASL, CCR7, VEGF and SOCS1 between tumor and normal group, IDO showed a reduction in tumor tissues compared to control samples (Fig. 7a). Regarding tumor stages (Fig. 7b), the results showed an increased expression of IDO in stage III compared to control group. An increased level of FASL was observed in association with tumor progression, although, at the first and last stage of breast cancer samples there was a decrease compared to control tissue. The expression level of SOCS1 in stage II was higher than all other groups. In addition, the data showed that VEGF was increased in stage II and III compared to stage I. The expression of CCR7 was not significantly different between stages of breast cancer and normal (Fig. 7b).

## Discussion

In this study, the gene expression profiling of four subsets of T CD4^+^ cells was investigated in the four different stages of breast cancer. We documented the presence of a significantly lower expression of Th1 and Th17 subset markers in the biopsies of breast cancer patients compared to control samples, however, a difference in the amount of Th2 or Treg cells between tumors and control groups was not seen. In this study, decreased infiltration of Th1 lymphocytes were shown in the microenvironment of advanced stages of breast cancer. The lowest expression of Th1 subset specific genes, including T-bet, IFN-γ and IL-12 were seen in stage IV. The cytokine IL-12, which stimulates Th1-dominant immunity, was shown to have strong anti-tumor activity against a variety of tumors, suggesting that Th1 cells may play an important role in tumor rejection [21]. IL-12 has effects on cytotoxic T lymphocytes and Th1 cells to produce IFN-γ which inhibits tumor cell cycle [22]. Reduction in the Th1 subset is related to tumor progression; suggesting that it may be directly implicated in the mechanisms that allow the tumor to progress and metastasize.

In this study, we found that the Th17 markers STAT3 and RORC were significantly decreased in stage IV. However, previous studies have reported controversial results. For example, one study consistent with our study suggested that the infiltration of Th17 cells reduced in progressive stages of breast cancer [23] while another demonstrated Th17 accumulation in association with cancer progression [24]. In the current study, progression of breast cancer probably is due to the decreasing of Th1 and Th17 levels in the microenvironment.

We found no significant changes of Th2 genes expression in the different stages of breast cancer. Th2 cells secrete IL-4, IL-5, IL-10 and IL-13 cytokines which induce T cell anergy and loss of T cell-mediated cytotoxicity [25]. In vitro assays discovered that cancer cells could direct the tumor-infiltrated T cells toward the Th2 phenotype. For example, cancer cells promote the production of IL-4 and down-regulate the expression of IFN-γ in the tumor microenvironment [26]. In contrast, some studies provide the finding that Th2-derived cytokines (IL-4, IL-5, IL-10, and IL13) show anti-tumor activities in vivo that are as strong as the anti-tumor activities of Th1 cytokines [21]. Nevertheless, in the current study the Th2 accumulation in different stages of breast cancer and normal breast samples was similar. However, it is likely that more samples are required to survey Th2 presence. Similar to Th2 markers, we found no significant changes in the expression of Treg markers. Our data shows that in patients with hormone receptors (ER and PR positive), the expression of Th2 marker (GATA3) was significantly higher than ER and PR negative patients. Consistently, it has been reported that the number of suppressive T cells is lower in triple negative (ER^−^/PR^−^/Her2^−^) patients [27]. Therefore, we assume that higher number of Th2 marker in breast cancer patients may be considered as a poor prognostic marker.

Breast cancer has a high level of heterogeneity both intertumorally and in the microenvironment. The microenvironment itself displays a wide variation in tissue cellularity and hormone receptor status between individuals [28]. It has been shown that hormone receptor profile is associated with the type of infiltrated immune cells in the tumor microenvironment; more T cell infiltration and higher expression of T cell markers were observed in patients with hormone receptor negativity [29, 30]. Moreover, the degree of T cell infiltration and T cell functionality differ among the molecular subtypes of breast cancer [29]. Therefore, we assume that this large intertumor heterogeneity may be possible reasons as to why we could not identify any difference in the expression of Th2 and Treg markers.

Contradictory to the process of acute inflammation which leads to tumor rejection, chronic inflammation is related to tumor progression [31, 32]. The results of the current study showed that the amount of TNF-α increases in parallel with the disease stage. It has been reported that in murine model of breast cancer in which inflammation was induced by LPS (lipopolysaccharide), cancer cells metastasize progressively [33].

Furthermore, the suppressive condition of the tumor microenvironment is due to several inhibitory markers which are produced by tumor or immune cells. In agreement with our results, one study demonstrated that after injection of chemotherapeutic drugs the amount of some soluble apoptosis markers such as soluble FASL is increased in stage II and III breast cancer [34]. The higher expression of IDO was reported in stages II and III of breast cancer [35] similar to our study. VEGF plays a crucial role in angiogenesis which is necessary for tumor development [36, 37]. Based on our data, there was an increase in VEGF expression followed by a decrease once the tumor was established (i.e. Stage IV). Still, the accumulation of immune cells in tumor microenvironment and their role in breast cancer prognosis are controversial.

## Conclusions

In the present study, we concluded that an impaired Th1 and Th17 cell anti-tumor responses causing tumor progression and providing some justification for exogenous IL-12 therapy. On the other hand, anti-inhibitory markers could boost immune response of breast cancer patients and control tumor progression and high level of anti-inflammatory responses might be able to control tumor progression.

## Abbreviations

Th cells: T helper cells
Treg: regulatory T cells
TNF-α: tumor necrosis factor-α
VEGF: vascular endothelial growth factor
CCR7: C-C motif chemokine receptor 7
IL-6: interleukin 6
IDO: indoleamine 2,3-dioxygenase
SOCS1: suppressor of cytokine signaling 1
ER: estrogen receptor
PR: progesterone receptor
Her2: human epidermal growth factor receptor 2
IDC: invasive ductal carcinoma
ILC: invasive lobular carcinoma
IHC: immunohistochemistry
TFRC: transferrin receptor
ACTB: β-actin
RORC: RAR related orphan receptor C
STAT3: signal transducer and activator of transcription 3
FOXP3: forkhead box P3
CCL22: C-C motif chemokine ligand 22
CTLA-4: cytotoxic T-lymphocyte associated protein 4
